# Production of scaffold-free cell-based meat using cell sheet technology

**DOI:** 10.1038/s41538-022-00155-1

**Published:** 2022-09-03

**Authors:** Ryu-ichiro Tanaka, Katsuhisa Sakaguchi, Azumi Yoshida, Hironobu Takahashi, Yuji Haraguchi, Tatsuya Shimizu

**Affiliations:** 1 Institute of Advanced Biomedical Engineering and Science, TWIns, Tokyo Women’s Medical University, Tokyo, Japan; 2 Department of Integrative Bioscience and Biomedical Engineering, Graduate School of Advanced Science and Engineering, TWIns, Waseda University, Tokyo, Japan

**Keywords:** Biomaterials - cells, Tissues

## Abstract

In the production of cell-based meat, it is desirable to reduce animal-derived materials as much as possible to meet the challenges of sustainability. Here, we demonstrate the “cell sheet-based meat”: scaffold-free cell-based meat using cell sheet technology and characterize its texture and nutrients. Bovine myoblast cell sheets were prepared using temperature-responsive culture dishes (TRCDs) and 10 stacked cell sheets to fabricate three-dimensional tissue of 1.3–2.7 mm thickness. Hardness was increased by incubation on the TRCD and was further increased by boiling as is characteristic of natural meat. The wet weight percentage of total protein in the cell sheet was about half that of beef. In this method, large-sized items of cell sheet-based meat were also created by simply scaling up the TRCD. This method promises an environment-friendly food product.

## Introduction

The global demand for meat is increasing in tandem with the growth of the world’s population^1,2^. However, conventional meat production emits large amounts of gases which contribute to global warming. These arise from processes such as feed production and manure management, which together account for 14.5% of the total greenhouse gases^3^. Therefore, increasing meat production to meet demand is expected to have an even greater impact on the environment. In contrast, cell-based meats, which are meat substitutes made from cultured animal cells, are environmentally friendly^4–6^. Compared to livestock rearing, cell-based meat production is estimated to significantly reduce greenhouse gas emissions because it reduces land use, water use, and food-crop consumption^7,8^. Cell-based meats are key to addressing sustainability challenges.

By using a tissue engineering technique, 3-D tissues can be made from cells^9^. There are two main methods of creating 3-D tissue from cells: scaffold-based and scaffold-free^10,11^. The scaffold-based method reproduces the muscle fiber structure according to the shape of the formed scaffold and provides a meat-like texture owing to the hardness of the scaffold^12–14^. However, most of the scaffolds used in the reported studies were based on animal-derived proteins. Therefore, it is desirable to develop techniques that reduce the use of animal-derived materials as much as possible, for cost reduction and sustainable production^5,15^.

Cell sheet technology is one method used to create scaffold-free 3-D tissues^16^. In this method, cell sheets are prepared using temperature-responsive culture dishes (TRCDs). The TRCD’s culture surface is covalently bonded with temperature-responsive polymer: poly(*N*-isopropylacrylamide) (PIPAAm) using electron beam irradiation, which makes the surface is hydrophilic below 32 °C and hydrophobic at 37 °C^17,18^. This property allows cells to adhere to the dish surface and remain attached to each other at 37 °C, yet to detach from the dish surface while remaining attached to each other below 32 °C, thereby producing cell sheets. 3-D tissues with a thickness of several millimeters can be easily created by stacking and attaching multiple cell sheets to each other. The stacked cell sheets thus prepared are characterized by high cell density. Since the area of the cell sheet depends on the area of the TRCD, cell sheets having a large area can be easily produced using larger TRCDs. Using this method, various functional tissues of different function—such as skeletal muscle^19,20^, liver tissue^21,22^, and cardiac tissue^23–25^—have been produced in vitro with the aim of developing cell-based regenerative therapy and screening models for drug discovery. This method is suitable also for producing cell-based meat because it is relatively easy to scale up and can be scaffold-free. Therefore, we aimed to create scaffold-free cell-based meat by using cell sheet technology (cell sheet-based meat) and to characterize the texture and nutrients.

## Results

### Dimensional and structural characteristics of the bovine myoblast cell sheets

**Fig. 1:**
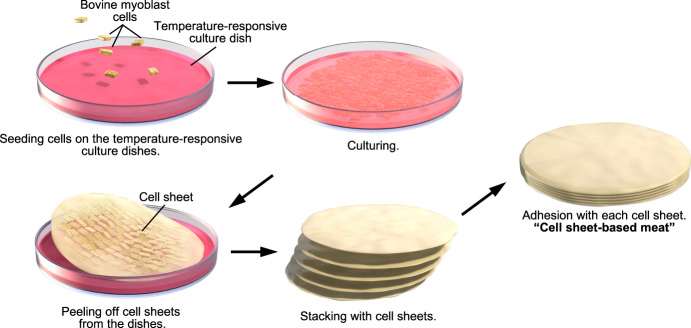
Production of cell sheet-based meat.

**Fig. 2:**
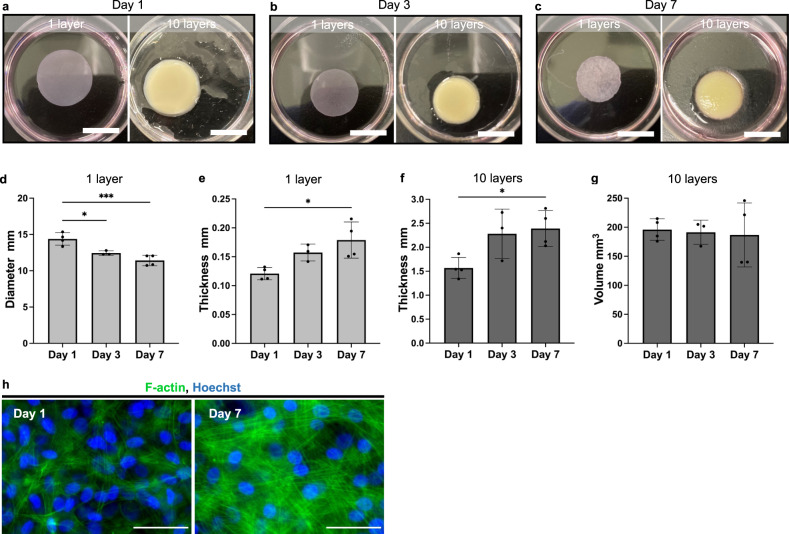
Dimensional and structural characteristics of the bovine myoblast cell sheets. **a**–**c** One and 10-layered-bovine myoblast cell sheets. **a** day 1 of culture. **b** day 3 of culture. **c** day 7 of culture. Scale bar: 1 cm. **d** Diameter and (**e**) thickness of the individual bovine myoblast cell sheets (*n* = 4). **f** Thickness and (**g**) volume of the 10-layered-bovine myoblast cell sheets (*n* = 4). **h** Representative immunofluorescent images of bovine myoblast cell cytoskeleton (Green: F-actin, blue: Hoechst). Left: day 1 of culture. Right: day 7 of culture. Scale bar: 50 μm. In (**d**–**g**) the data points represent individual values and error bars represent SD. *P < 0.05, **P < 0.01 was considered significant in the analysis between groups using one-way ANOVA, with Tukey’s HSD.

In this study, we prepared cell sheets using bovine myoblast cells and stacked 10 layers of these sheets to create cell-based meat (Fig. 1). The bovine myoblast cells were seeded at a density of 5 × 10^6^ cells in a 3.5 cm diameter temperature-responsive culture dish and cultured in an incubator at 37 °C for 1, 3, or 7 days; the prepared cell sheets were then detached from the culture dishes by further incubation at 20 °C (Fig. 2a). Ten sheets of the bovine myoblast cells were stacked to form a 3-D tissue (Fig. 2a–c). The diameters of the prepared sheets on days 1, 3, and 7 were 14.7 ± 0.6, 12.2 ± 0.7, and 11.2 ± 0.7 mm, respectively (Fig. 2d). The diameter of the bovine myoblast cells sheets was significantly smaller on days 3 and 7 compared to that on day 1. The thicknesses of the individual sheets were 0.12 ± 0.01, 0.14 ± 0.01, and 0.17 ± 0.04 mm (Fig. 2e), while those of the 10-layered sheets were 1.43 ± 0.11, 2.04 ± 0.01, and 2.28 ± 0.39 mm (Fig. 2f) on days 1, 3, and 7, respectively. The volumes of the 10-layered tissue construct on days 1, 3, and 7, calculated from the area and thickness values, were 245.9 ± 35.0, 238.4 ± 50.6, and 229.2 ± 70.8 mm^3^, respectively, and did not differ significantly between each other (Fig. 2g). To confirm the cytoskeleton of the bovine myoblast cell sheets, the cells were seeded into normal culture dishes under the same conditions and seeding density as those used for cell sheet preparation and were cultured for 1 or 7 days. F-actin staining with phalloidin (Fig. 2h) revealed that the amount of F-actin was higher on day 7 of culture compared to that of day 1.

Next, we investigated whether TRCDs can be reused. This was done by detaching the bovine myoblast cell sheet from the used TRCD, washing and sterilizing, and seeding bovine myoblast cells again. The 1-day cultured bovine myoblast cell sheet was possible to be detached again from the used TRCD. Supplementary Fig. 1 shows bovine myoblast cell sheets prepared using new or used TRCDs. Three TRCDs were used simultaneously for the experiment, and were all found to reusable. Thus, it was confirmed that the TRCDs could be reused up to two times.

### Histological analysis of bovine myoblast cell sheets

**Fig. 3:**
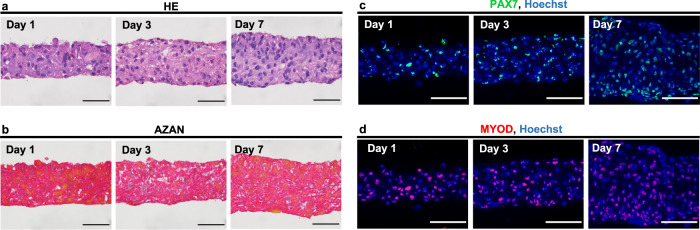
Histological analysis of a bovine myoblast cell sheet. **a** HE-stained sections of bovine myoblast cell sheets. **b** AZAN-stained sections of bovine myoblast cell sheets. **c** PAX7 and **d**, MYOD staining of the bovine myoblast cell sheets. Left: day 1 of culture, center: day 3 of culture, right: day 7 of culture. Scale bar: 1 cm.

Histological evaluation of the hematoxylin and eosin (HE)-stained sections showed that the thickness of the prepared bovine myoblast cell sheets increased with the number of culture days, with no significant structural differences (Fig. 3a). In addition, AZAN staining performed to confirm the presence or absence of collagen fibers (Fig. 3b) showed that most of the cells were stained red, and not blue, on day 7, indicating the absence of collagen fibers. Fluorescent immunostaining for PAX7 and MYOD, markers of myoblast cells, was also performed (Fig. 3c, d). Most of the cells were positive for PAX7 and MYOD, indicating most were myoblast cells.

### Texture profile analysis of stacked cell sheets and beef

**Fig. 4:**
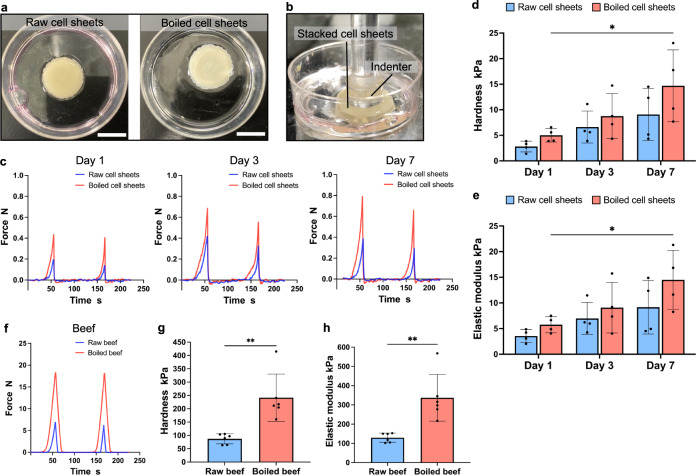
TPA of the stacked cell sheets and beef. **a** Left: raw cell sheet tissue. Right: boiled cell sheet tissue. Scale bar: 1 cm. **b** Image of the indenter pressing the stacked cell sheet. **c** TPA curves, (**d**) hardness, and (**e**) Elastic modulus of raw and boiled stacked cell sheets (*n* = 4). **f** TPA curves, (**g**) hardness, and (**h**) Elastic modulus of raw and boiled beef (*n* = 6). In (**d**, **e**), the error bars represent SD, and the data points represent individual values. *P < 0.05 was considered significant in the analysis between groups using two-way ANOVA, with Tukey’s HSD. In (**g**) and (**h**), the error bars represent SD, and the data points represent individual values. **P < 0.01 was considered significant in the analysis between paired *t* tests.

The 10-layered cell sheet-based meat was then subjected to texture tests on days 1, 3, and 7. The texture tests were performed on both raw cell sheets (Fig. 4a, left) and boiled cell sheets (Fig. 4a, right), which were prepared by heating the raw sheets in water at 85–90 °C for 90 s. The heating time was determined based on the time required for cooking commercial beef with a thickness of 1–2 mm. Supplementary Video 1 showed that boiled 10-layer cell sheet-based meat pieces were picked, cut, and pulled. For the texture test, a circular probe (10 mm diameter) was pressed twice into the stacked bovine myoblast cell sheets and the force exerted by the sheets was measured (Fig. 4b, Supplementary Video 2). Based on the texture profile analysis curves obtained (Fig. 4c), the maximum pressure (hardness) exerted during the first push was compared (Fig. 4d). The hardness tended to improve with the increase in the number of days of incubation, with a significant difference observed on days 1 (5.00 ± 1.31 kPa) and 7 (14.69 ± 7.03 kPa) for boiled cell sheets. The hardness of the boiled cell sheets also tended to be higher than that of raw tissue, and there was a significant difference between them on day 1 (raw: 2.80 ± 1.05 kPa, boiled: 5.00 ± 1.31 kPa) and day 7 (raw: 9.07 ± 5.11 kPa, boiled: 14.69 ± 7.03 kPa). The elastic modulus (Fig. 4e). As with hardness, the elastic modulus tended to increase with the number of incubation days, and also tended to increase with heating compared to that before heating. The same method was then used to test the texture of commercial beef with a thickness of 1–2 mm. The TPA curves obtained from the texture measurements (Fig. 4f) showed that the hardness of the raw and boiled beef was 87.19 ± 18.93 and 241.55 ± 88.88 kPa, respectively, and the elastic modulus was 13.22 ± 1.28 and 22.66 ± 3.99 kPa, respectively. Compared with the sheet tissue of day 7, which had the highest hardness and modulus, the hardness of the raw and boiled beef was 9.6 and 16.5 times higher, respectively, while the elastic modulus was 14.2 and 23.22 times higher.

Cohesiveness, springiness, chewiness, brittleness, and adhesiveness were then evaluated using the analysis method of Szczeniak^26^ (Supplementary Fig. 2a). Cohesiveness was expressed as the ratio of the energy required for the first push to that required for the second push (Supplementary Fig. 2b). The cohesiveness of the stacked bovine myoblast cell sheets on days 1, 3, and 7 did not differ significantly between each other in both raw and boiled tissues, while a significant difference was observed between the cohesiveness of beef and stacked bovine myoblast cell sheets, especially after heating. Springiness was expressed as the ratio of the distance from the point at which the force was detected at the beginning of the push to the maximum push position (Supplementary Fig. 2c). The springiness of the stacked raw sheets tended to decrease as the number of culture days increased; however, the difference was not significant. On boiling, the springiness of the stacked sheets decreased on days 1 and 3 and increased on day 7; this difference was also not significant. Chewiness was calculated by multiplying the hardness, cohesiveness, and springiness of the stacked tissues and beef (Supplementary Fig. 2d). The chewiness tended to increase with the number of culture days. Brittleness represents the amount of force drop at which the sample is broken during the pressing process (Supplementary Fig. 2e). The brittleness decreased with the increase in the number of culture days and tended to be less detectable in the boiled tissues, with the boiled tissue of day 7 showing no brittleness. Moreover, the brittleness of commercial beef was lower than that of the stacked sheets and tended to be smaller after heating compared to raw beef. Adhesiveness represents the energy exerted per volume of the sample in the negative direction when the indentation jig is pulled up (Supplementary Fig. 2f). The adhesiveness of the stacked sheets did not differ significantly between the different days of culture or on heating; however, the values for commercial beef tended to decrease on heating.

### Nutritional analysis of cell sheets and beef

**Fig. 5:**
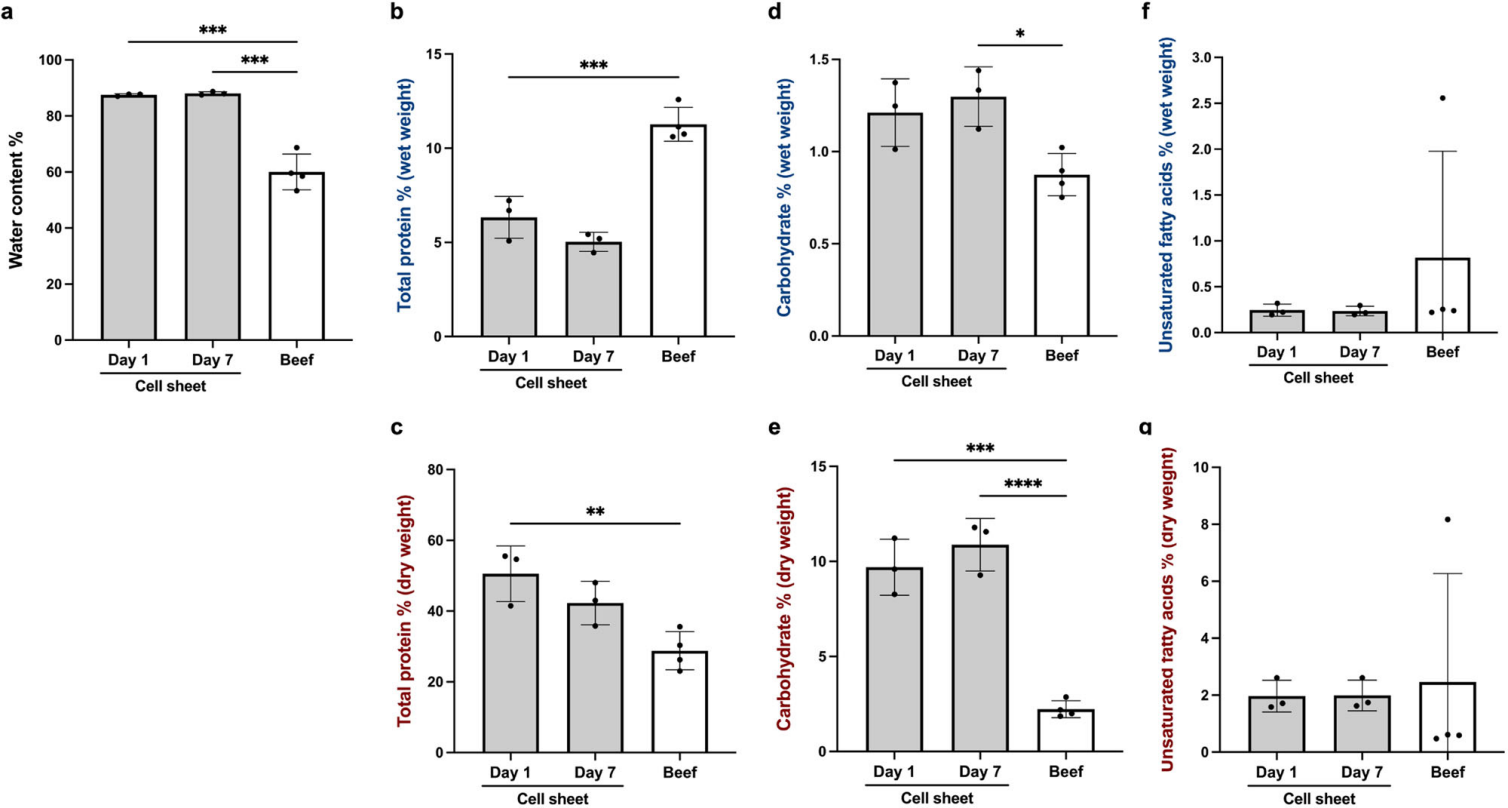
Nutritional analysis of cell sheets and beef. **a** Water content of cell sheets and beef. **b** Total protein in wet weight. **c** Total protein in dry weight. **d** Carbohydrate in wet weight. **e** Carbohydrate in dry weight. **f** Unsaturated fatty acids in wet weight. **g** Unsaturated fatty acids in dry weight. In (**b**–**g**), the data points represent individual values (Cell sheet: *n* = 3, Beef: *n* = 4), and the error bars represent SD. **P < 0.01, ***P = 0.0001, ****P < 0.0001 was considered significant in the analysis between groups using one-way ANOVA, with Tukey’s HSD.

The nutrients contained in the stacked bovine myoblast cell sheets on day 1 and day 7 of culture were compared with those in beef, and the water content of bovine myoblast cell sheets and beef was measured (Fig. 5a). The water content of bovine myoblast cell sheets at day 1 of culture was 87.5 ± 0.4% while on day 7 it was 88.1 ± 0.6%; that of beef was measured at 60.0 ± 6.4%. The water content of bovine myoblast cell sheets varied only slightly with the number of culture days and was more than 20% higher than that of beef.

The Bradford method was used to determine the percentage of total protein (Fig. 5b, c). The wet weight percentage of total protein in the bovine myoblast cell sheets was 6.3 ± 1.1% and 5.0 ± 0.5% on days 1 and 7, respectively, while that of beef was 11.3 ± 0.9%. The dry weight percentage of total protein in the bovine myoblast cell sheets was 50.6 ± 7.9% and 42.3 ± 6.1% on days 1 and 7, respectively, and that of beef was 28.8 ± 5.4%, significantly lower than that of the bovine myoblast cell sheet on day 1 of culture. Because of the high water content of bovine myoblast cell sheets, the percentage of total protein is lower than that of beef when compared by wet weight, but higher than that of beef when compared by dry weight.

The phenol-sulfuric acid method was then used to compare the percentage of carbohydrates (Fig. 5d, e). The wet weight percentage of carbohydrates in the bovine myoblast cell sheets was 1.2 ± 0.2% and 1.3 ± 0.2% on days 1 and 7, respectively, with no significant difference between the number of days, while in beef it was 0.9 ± 0.1 %, slightly but significantly below that of the bovine myoblast cell sheets. The dry weight percentage of carbohydrates in the bovine myoblast cell sheets was 9.7 ± 1.5% and 10.9 ± 1.4% on days 1 and 7, respectively, with no significant difference between the number of days of culture, while that in beef was 2.2 ± 0.4%, which was significantly lower than that in the bovine myoblast cell sheets.

The vanillin-sulfate method was then used to compare the percentage of unsaturated fatty acids (Fig. 5f, g). The wet percentage of unsaturated fatty acids in the myoblast cell sheets was 0.2 ± 0.1 and 0.2 ± 0.1% on days 1 and 7, respectively, while that in beef was 0.8 ± 1.2%. The dry percentage of unsaturated fatty acids in the myoblast cell sheets was 2.0 ± 0.6 and 2.0 ± 0.5% on days 1 and 7, respectively, with no significant difference between the number of days of culture, while that in beef was 2.5 ± 3.8%, with a large variation.

### Production of large-sized cell sheet-based meat

To make large-sized cell sheet-based meat items, bovine myoblast cell sheets were prepared using 10 cm diameter TRCDs (Supplementary Fig. 3a). Twelve bovine myoblast cell sheets were stacked to produce cell sheet-based meat (Supplementary Fig. 3b). Finally, the large-sized cell sheet-based meat item was colored with red food coloring and served with small tomatoes and watercress as a demonstration of cooking (Supplementary Fig. 3c). The bovine myoblast cell sheets shown in Supplementary Fig. 3 were fixed in 4% paraformaldehyde (PFA).

## Discussion

Reducing animal-derived materials as much as possible is important to achieve sustainable production of cell-based meat. In this study, we demonstrated to produce scaffold-free cell-based meat by using cell sheet technology and clarified the characteristics of the cell sheet-based meat. The prepared cell sheet-based meat comprised a high density of bovine myoblast cells, prior to the stage of differentiation into myofibers for understanding the basic characteristics.

The diameter and thickness of the bovine myoblast cell sheets decreased and increased, respectively, after detachment, with the increase in the duration of culture (Fig. 2b, c). This shrinkage after detachment has also been observed in other cell types^27,28^, and it has been reported that this is because of the cell traction force exerted by actin filaments towards the center of the adherent cells, and that this force increases with time after seeding^29,30^. In other hand, extending the culture period of cells on the TRCD did not change the volume (Fig. 2g). The reason why the cells did not increase after 7 days of culture on the TRCDs is that there was no space for cell growth because of the overconfluent state (5.5 × 10^5^ cells/cm^2^). No or low cell proliferation on the TRCD is not a significant problem, as our method proposes separating the stages responsible for increasing the cell growth and constructing 3-D tissues as two distinct stages. This study focuses on developing a method for constructing 3-D tissue using TRCD, rather than for increasing the cell growth.

The hardness of the stacked bovine myoblast sheets increased with the extend in the culture days. Previous studies on cell spheroid have reported similar results^31,32^. To investigate the reason for this increase in hardness, HE and AZAN staining were performed. However, there was no significant difference in HE-stained cell sections after different culture durations, and no collagen fibers were observed at any time during the culture duration (Fig. 3a, b). However, the amount of F-actin tended to be higher in the cultured bovine myoblasts (Fig. 2h). It has been previously reported that actin filaments in cells affect hardness^33^. Therefore, in this study, the hardness increase of cell sheets seems to be due to the increase of F-actin expression. On the other hand, collagen secretion is an effective method used to engineer texture which can be achieved with cytokine^34^ or mechanical stimulation^35^. In addition, it is possible to further improve texture via long-term culture, manipulation of myoblast orientation, myotube formation^36^, and electrical stimulation. The ability of natural meat to increase its hardness upon heating was also confirmed in the stacked bovine myoblast cell sheets. It is considered that the process of intracellular protein denaturation and aggregation observed in meat on heating may have contributed to the improvement in the hardness of the stacked bovine myoblast cell sheets^37,38^.

Nutrients of bovine myoblast cell sheets and beef were compared. In wet weight, bovine myoblast cell sheets contain only 5–6% protein due to their high water content (Fig. 5a, b). However, in the case of dry weight, it was significantly higher than that in beef (Fig. 5c). The number of culture days was not related to the protein content of bovine myoblast cell sheets. Because weighed the bovine myoblast cell sheets were washed with phosphate-buffered saline (PBS) and the beef stored in air, wet weight comparisons are not exactly under the same conditions. In order to improve protein content, it will be necessary to produce cell sheet-based meat containing myotubes. Characteristically, the percentage of carbohydrates in bovine myoblast cell sheets was significantly higher than in beef. It is thought that glycogen accounts for the most of carbohydrates in the cell sheet. In this experiment, cell sheets were cultured in a high-glucose (4500 mg/L) medium; therefore, the percentage of carbohydrate in cell sheets is higher than that in beef. It may be possible to reduce stored glycogen and make healthier cultured meat by incubating cell sheets in a lower glucose medium. The unsaturated fatty acids in the bovine myoblast cell sheets are part of the phospholipids that make up the cell membrane^39,40^. The value of the percentage of unsaturated fatty acids varied widely because the percentage of fat in beef varied depending on cut of meat and individual differences.

We estimated the cell growth rate and the number of culture days required to produce 1 kg of cell-based meat from 1 g of bovine cheek muscle myoblast cells. 6–15 × 10^6^ cells can be obtained from 1 g of bovine cheek muscle using our method, and 2.9 × 10^11^ cells are required to produce 1 kg of cell-based meat if the cells don’t enlarge^41^. Thus, to produce 1 kg of meat from 1 g of meat, 2–5 × 10^4^-fold multiplication would be required. In this study, bovine myoblast cells were grown in culture in uncoated 10 cm culture dishes. Doubling time of bovine myoblasts under these culture conditions is 2–3 days. Thus, the time required to grow (0.5–2) × 10^5^-fold myoblast cells taken from 1 g, to prepare 1 kg worth of cells, is approximately 29–47 days.

We used some disposable plastic products in this study, but plastic materials need to be used less to reduce environmental impact and avoid fragment contamination in food production. In contrast to plastic materials, metal and glass materials can be re-molded to reuse materials and reduce environmental impact. In addition, metal materials have higher wear resistance than plastic materials, thus reducing the risk of fragment contamination of food products. It is possible to culture cells on metal or glass surfaces^42–44^. Since PIPAAm has been reported to graft onto metal and glass, we believe that production of TRCDs based on these materials may be possible^45–47^. Based on the above, we believe that it is possible to change the equipment in this study from plastic materials to metal or glass materials in the future.

Regarding cell sheet safety, various types of cell sheets have already been clinically transplanted in accordance with medical regulations. Although the number of transplanted cell sheets is limited in clinical application, large numbers of cell sheets might be consumed as food. Therefore, we should apply standard food regulations, including repeated dose oral toxicity experiments, to cell sheet-based meat to ensure its safety.

In this study, we showed that scaffold-free cell-based meat can be produced using cell sheet technology. Our findings demonstrate the feasibility of producing scaffold-free cultured meat using cell sheet technology, and scale-up. This method will contribute to the further development of sustainable food production technology.

## Methods

### Isolation of bovine myoblast cells

Isolation of bovine myoblast cells was performed as previously described^36^. Briefly, bovine myoblast cells were isolated from bovine cheek muscles. The bovine cheek muscles were minced with a scalpel. Minced muscles were transferred to a 50 ml centrifuge tube and soaked in Hanks’ balanced solution (Fujifilm Wako Pure Chemicals, 084-08345) with pronase (Sigma-Aldrich, 7433). The 50 ml tube was incubated at 37 °C in a water bath for 1 h with shaking. After incubation, the contents were added to Dulbecco’s modified Eagle’s medium (DMEM, Fujifilm Wako Pure Chemicals, 043-30085) supplemented with 10% fetal bovine serum (FBS, Gibco, 10270-106) and 1% Penicillin-Streptomycin-Amphotericin B Suspension (Fujifilm Wako Pure Chemicals, 161-23181). This suspension was passed through a 40 μm cell strainer (Falcon, 352340) and centrifuged at 1000 × *g* for 10 min. The supernatant was removed, and the remaining precipitate was added to DMEM supplemented with 10% FBS, 1% P/S and 10 ng/ml basic fibroblast growth factor (Fiblast Spray, Kaken Pharmaceutical). The cells were then seeded into 10 cm culture dishes (Greiner Bio-One) coated with laminin-511 (Easy iMatrix-511, Matrixome, 892018). The laminin-511 of the culture dishes was achieved by spreading Easy iMatrix-511 on the bottom of the dish and incubating at 37 °C for at least 1 h. The medium was changed every 2–3 days. Passaging was performed when the cells became confluent on day 6 or 7 after seeding and the bovine myoblast cells were collected.

### Preparation of bovine myoblast cell sheets

Bovine myoblast cells were cultured by passaging at 6- or 7-day intervals in DMEM with 10% FBS, 1% Penicillin-Streptomycin (P/S, Fujifilm Wako Pure Chemicals, 168-23191) in 10 cm culture dishes. The bovine myoblast cells were cultured in TRCDs to prepare the cell sheets. The cells were seeded at 5 × 10^6^ cells/dish in 3.5 cm TRCDs, CellSeed, CS3017), coated with laminin-511 (Easy iMatrix-511, Matrixome, 892018), along with 2 ml of DMEM supplemented with 10% FBS and 1% P/S. Laminin-511 coating was performed by spreading Easy iMatrix-511 on the bottom of the dish and incubating at 37 °C for at least 1 h. The cells were then cultured in a CO_2_ incubator at 37 °C. The medium was changed on the day after seeding and again on day 4. Bovine myoblast cell sheets were detached from the TRCDs after incubating at 20 °C for 30 min in an incubator on the day after seeding day 1, day 3, or day 7.

Large-sized bovine myoblast cell sheets were prepared using 10 cm TRCDs (CellSeed, CS3017). The cells were seeded at 3 × 10^7^ cells/dish in 10 cm TRCDs along with 10 ml of DMEM supplemented with 10% FBS and 1% P/S. After 1 day culturing in a CO_2_ incubator at 37 °C, followed by 20 °C for 30 min, bovine myoblast cell sheets were detached from the TRCDs.

### Preparation of stacked cell sheets

For preparing the 3-D tissues, two bovine myoblast cell sheets were placed one on top of the other on a culture dish and allowed to stand in an incubator at 37 °C for 15 min to adhere. Another cell sheet was spread on top of the adhered bovine myoblast cell sheets and the above process was repeated. The procedure was repeated until a 3-D tissue consisting of 10 bovine myoblast cell sheet layers was prepared.

The detached large-sized bovine myoblast cell sheets were stacked in the same method and 8, 10 (Supplementary Fig. 3c) or 12 (Supplementary Fig. 3a) bovine myoblast cell sheet layers were prepared. After stacking, the large-sized cell sheet-based meat was fixed with 4% PFA. In Supplementary Fig. 3c, the cell sheet-based meat was colored with red food coloring (Kyoritsu foods, 1100460).

### Reuse temperature-responsive culture dishes

After 1-day cultured bovine myoblast cell sheets were prepared as described above, used TRCDs were sonicated in ultrapure water for 20 min by using an ultrasonic cleaner (ASONE, ASU-6D). Ultrasonic washing was performed while cooling below 20 °C with ice to remove proteins adhered to the surface. To sterilize the dishes after washing, 70% ethanol was placed in the TRCDs and allowed to stand for several minutes, after which the 70% ethanol was rinsed off in PBS. Bovine myoblast cell sheets were prepared again using washed TRCDs.

### Diameter, thickness, and volume measurements of the bovine myoblast cell sheets

The diameter of the bovine myoblast cell sheets was determined from the area of the sheets calculated using Image J software as follows: Diameter = $2 \times \sqrt {{\rm{Area}}/\pi }$. The thickness of the individual and stacked bovine myoblast cell sheets were measured using the cross-sectional images obtained by optical coherence tomography (Santec, IVS-2000). Three cross-sectional images were taken from one sample. For each of three images, the average of 5 thickness measurements (using ImageJ software) was calculated, then the average of the three images was used as the thickness value of the sample. The volume of the 10-layered-bovine myoblast cell sheets was determined as follows: volume = $\pi ({\rm{Diameter}}/2)^2 \times {\rm{Thickness}}$.

### TPA

The texture of the stacked bovine myoblast cell sheets and commercial beef was measured using a texture analyzer (Japan Instrumentation System, TEX-100N). A sample on a dish was placed in the texture analyzer, and the dish surface was set as point 0. A circular probe with a diameter of 10 mm was pressed twice into the sample at a speed of 0.04 m/s from heights of 0.3–2 mm to measure the force exerted by the samples. Hardness was determined as the maximum value of stress. The stress was calculated by dividing the measured force by the area of the probe. Cohesiveness, springiness, chewiness, brittleness, and adhesiveness were evaluated based on the analysis method defined by Szczeniak^26^ (Supplementary Fig. 2a). Elastic modulus is the slope of the approximate line obtained from the strain-stress plot.

### Histological analysis

The bovine myoblast cell sheets were fixed with 4% paraformaldehyde (Muto Pure Chemicals, 33111) overnight at 4 °C, and paraffin-embedded sections with a thickness of 5 μm were prepared. HE staining and AZAN staining were performed as previously described. The sections were photographed using an optical microscope. PAX7 and MYOD were incubated with 1:200 dilution of anti-PAX7 (Abcam, ab34360) and anti-MYOD (Santa Cruz, sc377460) at 4 °C overnight, and positive cells were detected. For the secondary antibody staining, a 1:500 dilution of Alexa Fluor 488 goat anti-rabbit IgG (H + L) (Invitrogen, A-11034) and Alexa Fluor 568 goat anti-mouse IgG (H + L) (Invitrogen, A-11031) were used for immunolabeling by overnight incubation at 4 °C.

### Phalloidin staining

Bovine myoblast cells were seeded in 96 well plates (Corning, 353072) coated with laminin-511 (Easy iMatrix-511, Matrixome, 892018) at a concentration of 5.2 × 10^5^ cells/cm^2^ and cultured for 1 or 7 days. Laminin-511 coating was performed by spreading Easy iMatrix-511 on the bottom of the dish and incubating at 37 °C for at least 1 h. The culture medium was changed on the day after seeding and again on day 4. The cultured bovine myoblast cells were fixed with 4% paraformaldehyde (Muto Pure Chemicals, 33111) for 20 min, washed three times with PBS, left to stand in PBS added with 0.1% Triton X-100 surfactant for 15 min, and then washed three times with PBS. The cells were then added with 100 μl of reagent (Abcam, ab176753) diluted 1000-fold in 1% BSA, and allowed to stand for 45 min, following which they were washed three times with PBS. Thereafter, 100 μl of Hoechst solution (Thermo Fisher Scientific, H3570) diluted 500-fold in PBS was added and the cells were allowed to stand for 15 min. The cells were then washed three times with PBS. The stained cells were observed by fluorescence microscopy.

### Nutritional analysis

The bovine myoblast cell sheets cultured in the temperature-responsive culture dishes were detached at day 1 or 7 of culture, placed in 1.5 ml tubes, and washed thrice with PBS. The PBS solution was removed after precipitating the cell sheets by centrifugation. Similarly, minced bovine cheek meat was placed in a 1.5 ml Eppendorf tube. The weight of the Eppendorf tube containing the cell sheets and bovine cheek meat was measured, and the wet weight was calculated by subtracting the weight of the Eppendorf tube. The cells were dried overnight by freeze-drying, and the dry weight was calculated. Thereafter, 1 ml of saline solution was added to each of the tubes containing the dried cell sheets and bovine cheek meat, and the samples were homogenized using an ultrasonic grinder (BIORUPTOR, Cosmo Bio), which was repeatedly switched on and off at 30-s intervals, for 30 min.

Proteins were quantified using the Bradford method. In brief, the homogenized samples were mixed with 2% sodium dodecyl sulfate solution in the ratio 3:1 and heated at 95 °C for 3 min. The samples were then cooled at 4 °C for 5 min and centrifuged at 3500 × *g* for 10 min. Ten microliters of the samples were placed in the wells of a 96 well plate and 300 μl of Bradford assay reagent (23246, Thermo Scientific) was added to each well and mixed. After incubation at room temperature for 10 min, the absorbance was measured at 595 nm using a spectrophotometer. The protein concentration of the cell sheets on days 1 and 7 were compared with that of the minced bovine cheek meat.

Carbohydrates were measured by the phenol-sulfuric acid method using an assay kit (STA-682, CELL BIOLABS). In brief, the homogenized sample solutions were centrifuged at 3500 × *g* for 10 min to remove debris. Thirty microliters of each sample was mixed with 150 µl of sulfuric acid in a 1.5 ml Eppendorf tube and heated at 90 °C for 15 min. After heating, the samples were transferred to a 96 well plate and the background was determined from the absorbance at 490 nm. Thereafter, 30 µl of 5% phenol solution was added to the samples and stirred using a rotary shaker for 30 min. The absorbance was measured at 490 nm, and a standard curve was prepared from the glucose standard to determine the carbohydrate concentration of the samples.

Unsaturated fatty acids were measured using the vanillin-sulfate method. In brief, lipids were extracted from the homogenized sample solutions using a lipid extraction kit (STA-612, CELL BIOLABS) and dissolved in dimethyl sulfoxide. Fifteen microliters of each sample was then mixed with 150 µl of sulfuric acid in a 1.5 ml Eppendorf tube and heated at 90 °C for 10 min. After heating, the samples were transferred to a 96 well plate and the absorbance was determined at 540 nm. Next, 100 μl of vanillin reagent was added to each well and incubated at 37 °C for 15 min, after which the absorbance was measured at 540 nm and a standard curve was prepared using the lipid standard to determine the unsaturated fatty acid concentration of the samples.

### Statistical analysis

One-way or two-way analysis of variance (ANOVA) was used for statistical analysis, and Tukey’s post-hoc test was used to compare multiple groups. Prism 9 (GraphPad) was used for the statistical analyses.

## Supplementary information

Supplemental Material

Supplementary Video 1

Supplementary Video 2

## Data Availability

The data that support the findings of this study are available upon reasonable request from the authors.

## References

[1] Cole MB, Augustin MA, Robertson MJ, Manners JM (2018). The science of food security. npj Sci. Food.

[2] FAO. *World Livestock 2011—Livestock in Food Security* (Food and Agriculture Organization of the United Nations—FAO, 2011).

[3] Gerber, P. J. et al. *Tackling Climate Change through Livestock—A Global Assessment of Emissions and Mitigation Opportunities* (Food and Agriculture Organization of the United Nations—FAO, 2013).

[4] Post MJ (2012). Cultured meat from stem cells: challenges and prospects. Meat Sci..

[5] Post MJ (2020). Scientific, sustainability and regulatory challenges of cultured meat. Nat. Food.

[6] Ben-Arye T, Levenberg S (2019). Tissue engineering for clean meat production. Front. Sustain. Food Syst..

[7] Tuomisto HL, Teixeira De Mattos MJ (2011). Environmental impacts of cultured meat production. Environ. Sci. Technol..

[8] Godfray, H. C. J. et al. Meat consumption, health, and the environment. *Science* **361**, eaam5324 (2018). 10.1126/science.aam5324 30026199

[9] Langer R, Vacanti JP (1993). Tissue engineering. Science.

[10] Khodabukus A, Prabhu N, Wang J, Bursac N (2018). In vitro tissue-engineered skeletal muscle models for studying muscle physiology and disease. Adv. Healthc. Mater..

[11] Moroni L (2018). Biofabrication: a guide to technology and terminology. Trends Biotechnol..

[12] Furuhashi M (2021). Formation of contractile 3D bovine muscle tissue for construction of millimetre-thick cultured steak. npj Sci. Food.

[13] Kang, D. H. et al. Engineered whole cut meat-like tissue by the assembly of cell fibers using tendon-gel integrated bioprinting. *Nat. Commun.* **12**, 5059 (2021). 10.1038/s41467-021-25236-9 PMC8385070 34429413

[14] MacQueen LA (2019). Muscle tissue engineering in fibrous gelatin: implications for meat analogs. npj Sci. Food.

[15] Ben-Arye T (2020). Textured soy protein scaffolds enable the generation of three-dimensional bovine skeletal muscle tissue for cell-based meat. Nat. Food.

[16] Yang J (2005). Cell sheet engineering: recreating tissues without biodegradable scaffolds. Biomaterials.

[17] Yamada N (1990). Thermo‐responsive polymeric surfaces; control of attachment and detachment of cultured cells. Die Makromol. Chem. Rapid Commun..

[18] Fukumori K (2010). Characterization of ultra-thin temperature-responsive polymer layer and its polymer thickness dependency on cell attachment/detachment properties. Macromol. Biosci..

[19] Takahashi H, Shimizu T, Okano T (2018). Engineered human contractile myofiber sheets as a platform for studies of skeletal muscle physiology. Sci. Rep..

[20] Takahashi H, Shimizu T, Nakayama M, Yamato M, Okano T (2013). The use of anisotropic cell sheets to control orientation during the self-organization of 3D muscle tissue. Biomaterials.

[21] Gao B (2020). In vitro production of human ballooned hepatocytes in a cell sheet-based three-dimensional model. Tissue Eng. A.

[22] Kim K, Ohashi K, Utoh R, Kano K, Okano T (2012). Preserved liver-specific functions of hepatocytes in 3D co-culture with endothelial cell sheets. Biomaterials.

[23] Shimizu T, Yamato M, Kikuchi A, Okano T (2003). Cell sheet engineering for myocardial tissue reconstruction. Biomaterials.

[24] Shimizu T (2006). Polysurgery of cell sheet grafts overcomes diffusion limits to produce thick, vascularized myocardial tissues. FASEB J..

[25] Sekiya S, Shimizu T, Yamato M, Kikuchi A, Okano T (2006). Bioengineered cardiac cell sheet grafts have intrinsic angiogenic potential. Biochemical Biophysical Res. Commun..

[26] Bourne, M. C. In *Food Texture and Viscosity* 118–198 (Elsevier, 1982). 10.1016/B978-0-12-119060-6.50009-5.

[27] Takagi S (2012). Cell shape regulation based on hepatocyte sheet engineering technologies. Cell Transplant..

[28] Haraguchi Y (2012). Fabrication of functional three-dimensional tissues by stacking cell sheets in vitro. Nat. Protoc..

[29] Lemmon CA, Chen CS, Romer LH (2009). Cell traction forces direct fibronectin matrix assembly. Biophysical J..

[30] Kuribayashi-Shigetomi K, Onoe H, Takeuchi S (2012). Cell origami: self-folding of three-dimensional cell-laden microstructures driven by cell traction force. PLoS ONE.

[31] Sarem M, Otto O, Tanaka S, Shastri VP (2019). Cell number in mesenchymal stem cell aggregates dictates cell stiffness and chondrogenesis. Stem Cell Res. Ther..

[32] Koudan EV (2020). Multiparametric analysis of tissue spheroids fabricated from different types of cells. Biotechnol. J..

[33] Chaudhuri O, Parekh SH, Fletcher DA (2007). Reversible stress softening of actin networks. Nature.

[34] Shi A, Hillege MMG, Wüst RCI, Wu G, Jaspers RT (2021). Synergistic short-term and long-term effects of TGF-β1 and 3 on collagen production in differentiating myoblasts. Biochem. Biophys. Res. Commun..

[35] Saito J (2021). Scaffold-free tissue-engineered arterial grafts derived from human skeletal myoblasts. Artif. Organs.

[36] Takahashi H, Yoshida A, Gao B, Yamanaka K, Shimizu T (2022). Harvest of quality-controlled bovine myogenic cells and biomimetic bovine muscle tissue engineering for sustainable meat production. Biomaterials.

[37] Tornberg E (2005). Effects of heat on meat proteins—implications on structure and quality of meat products. Meat Sci..

[38] Bertola NC, Bevilacqua AE, Zaritzky NE (1994). Heat treatment effect on texture changes and thermal denaturation of proteins in beef muscle. J. Food Process. Preservation.

[39] Harayama T, Riezman H (2018). Understanding the diversity of membrane lipid composition. Nat. Rev. Mol. Cell Biol..

[40] Széliová D (2020). What CHO is made of: variations in the biomass composition of Chinese hamster ovary cell lines. Metab. Eng..

[41] Allan, S. J., de Bank, P. A. & Ellis, M. J. Bioprocess design considerations for cultured meat production with a focus on the expansion bioreactor. *Front. Sustain. Food Syst.* **3**, 44 (2019).

[42] Rando TA, Blau HM (1997). Methods for myoblast transplantation. Methods Cell Biol..

[43] Groessner-Schreiber B, Tuan RS (1992). Enhanced extracellular matrix production and mineralization by osteoblasts cultured on titanium surfaces in vitro. J. Cell Sci..

[44] Bordji K (1996). Evaluation of the effect of three surface treatments on the biocompatibility of 316L stainless steel using human differentiated cells. Biomaterials.

[45] Smirnov EA (2013). Grafting of titanium dioxide microspheres with a temperature-responsive polymer via surface-initiated atom transfer radical polymerization without the use of silane coupling agents. Polym. Int..

[46] Liu YZ, Chen MS, Cheng CC, Chen SH, Chen JK (2017). Fabrication of device with poly(N-isopropylacrylamide)-b-ssDNA copolymer brush for resistivity study. J. Nanobiotechnol..

[47] Fukumori K (2009). Temperature-responsive glass coverslips with an ultrathin poly(N-isopropylacrylamide) layer. Acta Biomaterialia.

