# Supplementary material for: Production of scaffold-free cell-based meat using cell sheet technology
Source: NPJ Sci Food. 2022 Sep 3;6:41. doi: 10.1038/s41538-022-00155-1 (PMC9440907; doi:10.1038/s41538-022-00155-1)
Supplement: Supplementary file 1 — Supplemental Material [file 41538_2022_155_MOESM1_ESM.pdf]

**Supplemental Material**

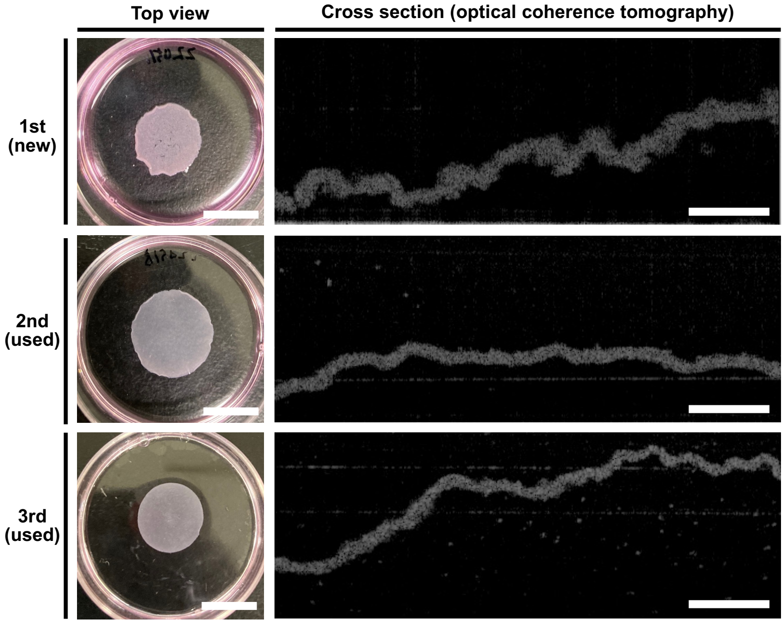


**Supplementary figure 1| Reuse of temperature responsive culture dishes (TRCDs).** Photographs showing top and cross section views of a bovine myoblast cell sheet after detaching from the TRCD. TRCDs were confirmed to be reusable two times. Used TRCDs were washed by ultrasonic cleaner and sterilized by 70% ethanol. Top view scale bar is 1 cm. Cross section scale bar is 500 µm.

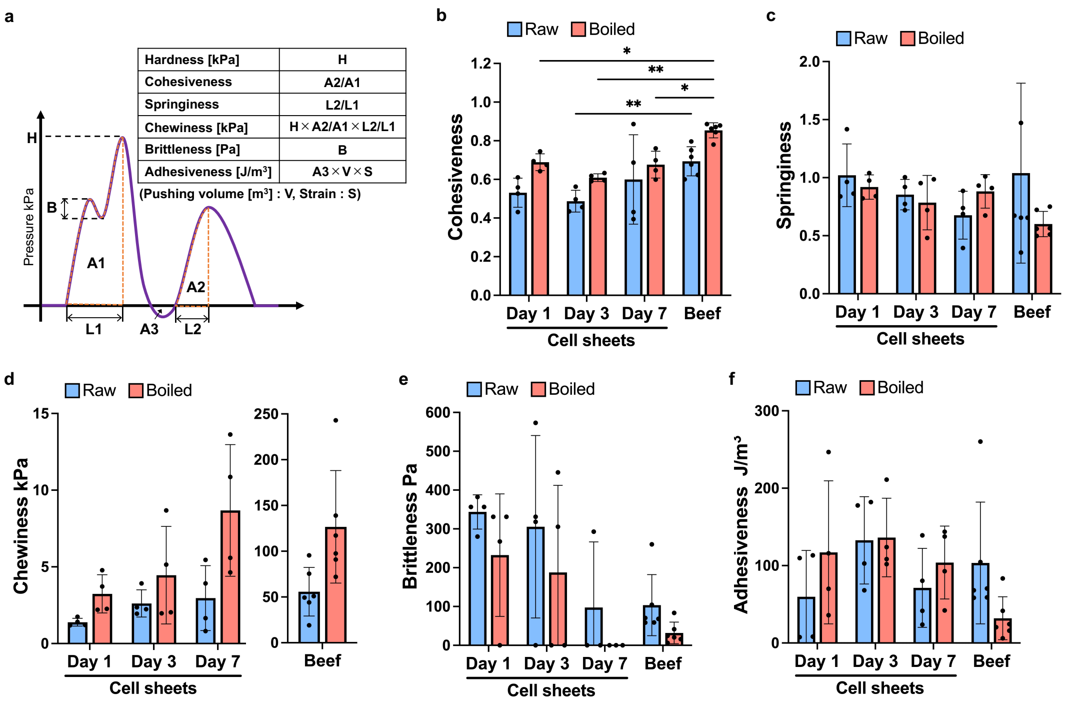


**Supplementary figure 2| Texture profile analysis (TPA) parameters.** **a**, Generalized TPA curve[26]. **b**, Cohesiveness. *P < 0.05, **P < 0.01 was considered significant in the analysis between groups using two-way ANOVA, with Tukey's HSD. **c**, Springiness. **d**, Cohesiveness. **e**, Brittleness. **f,** Adhesiveness. In **b-f**, the data points represent individual values (Cell sheet: n=3, Beef: n=4), and the error bars represent SD.

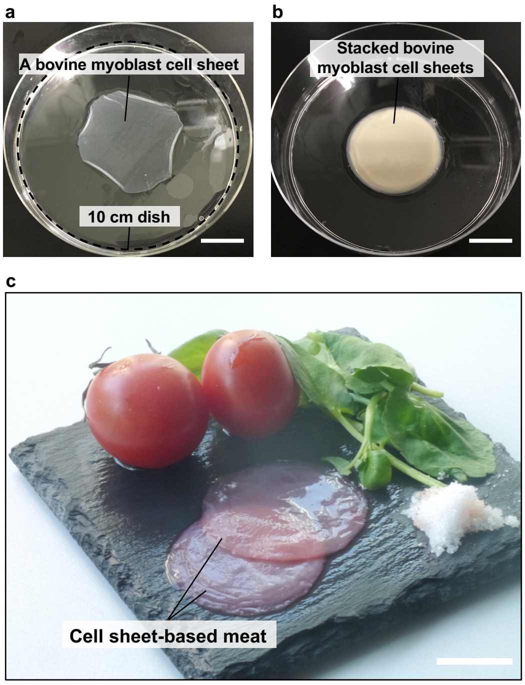


**Supplementary figure 3| Large-sized cell sheet-based meat.** **a**, a bovine myoblast cell sheet fabricated by using a 10 cm temperature-responsive culture dish. **b**, 12 layered bovine myoblast cell sheets fabricated by using 10 cm temperature-responsive culture dishes. **c**, Cell sheet-based meats, which are 8 layered and 10 layered cell bovine myoblast sheets, colored with red food coloring, and placed on a plate with tomato and watercress. The cell sheet-based meats were fixed with 4% PFA. In **a-c**, scale bar is 2 cm.

**Supplementary Video 1| Boiled cell sheet-based meat created by using 3.5 cm temperature-responsive culture dishes.**

**Supplementary Video 2| Texture profile analysis.**
